# Rapid and sensitive point-of-care detection of Orthopoxviruses by ABICAP immunofiltration

**DOI:** 10.1186/s12985-016-0665-5

**Published:** 2016-12-09

**Authors:** Daniel Stern, Victoria A. Olson, Scott K. Smith, Marko Pietraszczyk, Lilija Miller, Peter Miethe, Brigitte G. Dorner, Andreas Nitsche

**Affiliations:** 1 Centre for Biological Threats and Special Pathogens (ZBS), Robert Koch Institute, Seestrasse 10, 13353 Berlin, Germany; 2 Division of High-Consequence Pathogens and Pathology, Poxvirus and Rabies Branch, Centers for Disease Control and Prevention, Atlanta, GA USA; 3 Senova GmbH, Weimar, Germany; 4 FZMB GmbH, Bad Langensalza, Germany; 5 Novel Vaccination Strategies and Early Immune Responses, Paul-Ehrlich-Institut, Langen, Germany

**Keywords:** *Orthopoxvirus*, *Variola virus*, *Monkeypox virus*, *Cowpox virus*, *Vaccinia virus*, bioterrorism, Zoonosis, Point-of-care, Rapid detection, ABICAP

## Abstract

**Background:**

The rapid and reliable detection of infectious agents is one of the most challenging tasks in scenarios lacking well-equipped laboratory infrastructure, like diagnostics in rural areas of developing countries. Commercially available point-of-care diagnostic tests for emerging and rare diseases are particularly scarce.

**Results:**

In this work we present a point-of-care test for the detection of *Orthopoxviruses* (OPV). The OPV ABICAP assay detects down to 1 × 10^4^ plaque forming units/mL of OPV particles within 45 min. It can be applied to clinical material like skin crusts and detects all zoonotic OPV infecting humans, including *Vaccinia*, *Cowpox*, *Monkeypox*, and most importantly *Variola virus*.

**Conclusions:**

Given the high sensitivity and the ease of handling, the novel assay could be highly useful for on-site diagnostics of suspected *Monkeypox virus* infections in areas lacking proper laboratory infrastructure as well as rapid on-site testing of suspected bioterrorism samples.

**Electronic supplementary material:**

The online version of this article (doi:10.1186/s12985-016-0665-5) contains supplementary material, which is available to authorized users.

## Background

OPV belong to the *Poxviridae* family and are large and complex DNA viruses able to infect humans, causing severe diseases [1]. Despite the successful eradication of smallpox caused by *Variola virus* (VARV), zoonotic infections with still circulating OPVs, namely *Monkeypox virus* (MPXV), *Cowpox virus* (CPXV), and *Vaccinia virus* (VACV) [2], remain a threat to an increasing number of unvaccinated individuals [3]. Diagnostics and differentiation of OPV from other rash-inducing agents [4] are usually done by detection of either viral particles by negative staining electron microscopy or viral DNA by quantitative real-time PCR (qPCR), both requiring dedicated laboratories. However, rapid and highly sensitive point-of-care (POC) diagnostics are needed because of the rising incidence of MPXV infections in rural Africa [5, 6] as well as the risk of a potential deliberate release of *Variola virus* in the case of a bioterrorist attack [7, 8].

**Fig. 1:**
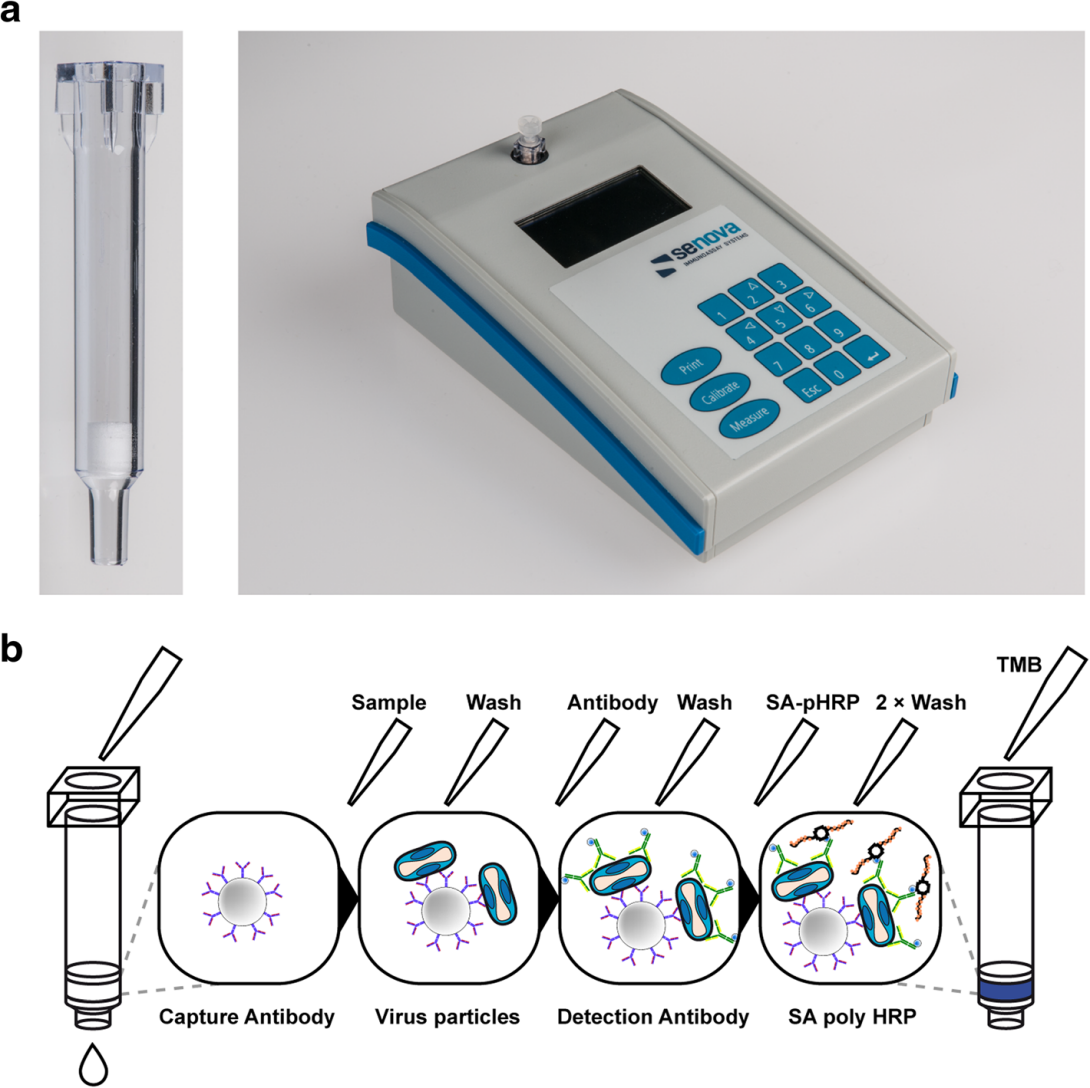
**a** ABICAP column with antibody-coated frit and handheld photometer device for readout at 525 nm (Senova, Weimar, Germany). **b** Schematic representation of the ABICAP assay procedure. Plastic columns with capture antibody-coated frits are filled with sample to capture the viral particles with the following steps: (1) Addition of pre-diluted sample material (sample dilution buffer), (2) washing with washing buffer, (3) addition of biotinylated detection antibody, (4) washing with washing buffer, (5) addition of streptavidin-(SA) PolyHRP, (6) two successive washing steps with washing and substrate buffer, (7) addition of precipitating TMB substrate, (8) final washing with substrate buffer, readout with a handheld photometer device

An ideal POC diagnostic system should be affordable, sensitive, specific, simple enough to perform by untrained persons, rapid and robust, free of sophisticated equipment, and deliverable to those who need it [9]. The ABICAP (Antibody Immuno Column for Analytical Processes) immunofiltration system fulfills these criteria [10]. The principle is based on a gravity-driven flow-through antigen capture ELISA (Fig. 1). Compared to established lateral flow assays or enzyme linked immunosorbent assays (ELISAs), the ABICAP assay offers several advantages. First, by immobilizing the capture antibody on a porous frit, the active surface coated with antibody is much larger as compared to the surface of an ELISA well. Thus the diffusion distance of the sample to the surface is greatly reduced, enabling much shorter incubation times (minutes instead of hours in the case of ELISA). Next, as the sample passes through the filter, enrichment out of larger sample volumes allows for a higher sensitivity of detection. Finally, as intermittent washing steps are possible, the integration of ELISA-like enzymatic amplification steps enables much higher sensitivity as compared to lateral flow assays. With an assay time of 45 min it is fast, the columns can be stored at ambient temperature, handling is simple, and the results can be read out by eye or a handheld photometric device.

In this work, we describe a rapid and sensitive detection system based on the ABICAP technology that reliably detects particles of different human-pathogenic OPV.

## Methods

### Antibodies and proteins

The diagnostic ABICAP is based on two monoclonal antibodies (mAbs) targeting the OPV surface protein A27: mAb A1/40 and mAb A3/710. Briefly, both antibodies bind to A27 with high affinity (A1/40: 2.3 nM; A3/710: 4.3 nM) and recognize non-overlapping linear epitopes located in close proximity to the N-terminal heparin-binding domain of A27 (A1/40: aa 24–38; A3/710: aa 13–27). Both antibodies have previously been selected in an antigen capture ELISA, representing the best combination to detect native VACV, CMLV, CPXV, ECTV, and MPXV. Production, purification, storage, and biotinylation of antibodies was performed as described previously [11]. The following reagent was obtained through the NIH Biodefense and Emerging Infections Research Resources Repository (BEI Resources), NIAID, NIH: *Vaccinia virus* (WR) rA27 (NR-2622) with a C-terminal histidine tag, recombinantly expressed from baculovirus.

### Viruses

The virus panel used for development and validation included VACV strain New York City Board of Health (NYCBOH, VR-1536™, ATCC/LGC Standards GmbH, Wesel, Germany) and CPXV strain GuWi [12]. *Camelpox virus* (CMLV) strain CP-19, *Ectromelia virus* (ECTV) strain Nü-1, and MPXV strain MSF6 were kindly provided by Prof. Dr. Hermann Meyer (Bundeswehr Institute of Microbiology, Munich, Germany). The OPV ABICAP detection system was additionally validated with two VARV strains, VARV Solaiman 1974 and VARV Niger 1969. Parapoxvirus (PPV) ORF D1701 was kindly provided by Achim Rziha (Friedrich-Loeffler-Institute, Tübingen, Germany) whereas herpes simplex virus 1 (HSV-1) was isolated in our lab from a patient. Propagation and titration of viruses on cell culture was done according to standard procedures [13]. Viruses were used as clarified supernatant (HSV, PPV, MPXV, and VARV) or as semi-purified viral particles by centrifugation through a 40% sucrose cushion [13]. All viruses were used natively except for MPXV which was heat-inactivated at 60 °C for 2 h. All work with live VARV was conducted within a biosafety level 4 laboratory in accordance to guidelines and approvals from the World Health Assembly Advisory Committee on Variola Virus Research.

For final validation, a panel containing inactivated (γ-irradiation) highly pathogenic viruses comprising *Yellow fever virus* strain 17D, *Ebola virus* strain Zaire, *Marburg virus*, VACV, and MPXV in Dulbecco’s Modified Eagle Medium was kindly provided by the P.R.O.B.E. consortium. Viruses in this panel have been quantified by quantitative real-time PCR using published assays [14, 15] and were investigated in a blinded manner to test the suitability of the ABICAP assay to discern highly pathogenic viruses in a bioterrorist setting.

### Clinical sample material

Clinical sample material tested in this work had been sent to the Robert Koch Institute for poxvirus diagnostics. Crust material and surface swabs were delivered dry and suspended in 500 μL of phosphate buffered saline (PBS, pH 7.4) without magnesium and calcium. Surface swabs were thoroughly mixed on a vortexer while crusts were homogenized in a Fastprep 24 homogenizer (MP Biomedicals, Eschwege, Germany) after addition of Precellys® ceramic beads (1.4 mm; PEQLAB, Erlangen, Germany), and DNA was prepared from 200 μL of sample using the Qiagen Blood and Tissues Kit (Qiagen, Hilden, Germany) according the manufacturer’s recommendations. Quantitative real-time PCR for OPV diagnostics was performed on 5 μL of isolated DNA, using rpo18 as target as published previously [16]. The number of genome equivalents (GE) contained in the samples was estimated based on the published calibration curve of the rpo18 assay. To test the specificity of the ABICAP assay, clinical samples negative in the OPV PCR were also tested. For differential diagnostics, different PCR assays targeting *Molluscipox virus* [17], *Parapox virus* [18], *Myxoma virus* [19] were tested on isolated DNA from homogenized crusts or swabs. Homogenized samples in PBS were tested in the ABICAP assay after dilution in UCBS casein buffer (SDT, Baesweiler, Germany).

### OPV ABICAP assay

The preparation of ABICAP columns was done as described before [10], using mAb A1/40 immobilized on polyethylene filter frits contained inside the ABICAP columns and biotinylated mAb A3/710 as the detection antibody.

To this aim, polyethylene filter frits (Type 180; Porex, Aachen, Germany) used for the coating of the capture antibodies were activated by successive 10-min washes with 96% ethanol, 50% ethanol, and 3 × coating buffer (0.1 M NaHCO_3_/ Na_2_CO_3_, pH 9.5). All activating and coating steps were done at room temperature under constant stirring and vacuum. The frits were then coated with 7.5 μg/frit of mAb A1/40 in coating buffer (75 μg/mL) overnight and blocked for 25 min with PBS-T BND (PBS + 0.05% Tween-20 + 0.05% BND [5-bromo-5-nitro-1,3-dioxane; SDT, Baesweiler, Germany]) + 0.2% bovine serum albumin (BSA; Carl Roth, Karlsruhe, Germany). Finally, frits were prepared for drying by 1 h of incubation with PBS-T BND + 1% BSA + 5% saccharose and air-dried at 35 °C for 30 min in a fluid bed dryer (FBD2000; Endecotts, London, UK). The columns were equipped with antibody-coated frits between two preblocked separation frits (Type 187; Porex) and stored dry at room temperature until further use.

The principle of ABICAP™ immunofiltration assay (Antibody Immuno Column for Analytical Processes, Senova GmbH, Weimar, Germany) is shown in Fig. 1. 500 μL of pre-diluted sample material (UCBS casein buffer, SDT) were applied per column and incubated for 6 min. After a washing step with 750 μL of washing buffer (PBS-T BND + 0.1% BSA), 500 μL of biotinylated mAb A3/710–20 (in AA1-buffer [SDT]) were added and incubated for 6 min. Columns were washed again and incubated with 500 μL of streptavidin-PolyHRP 40 (SA-pHRP; in SA1-buffer, both SDT) for 6 min. Two washing steps later (1 × 750 μL of washing buffer, 1 × 750 μL of substrate buffer [phosphate/citrate buffer, pH 5.0]) 500 μL of precipitating TMB substrate (epTMB; SDT) were added for 6 min. Finally, columns were washed with 750 μL of substrate buffer, and the extinctions were read at 525 nm with a handheld photometer device (Senova; Fig. 1).

To allow for maximum sensitivity of detection at low background, both SA-pHRP and biotinylated detection antibodies were titrated (0.1, 0.5, 1, and 5 μg/mL) in the absence of antigen. For titration of SA-pHRP, the protocol was performed as described, but instead of biotinylated detection antibody AA1-buffer only was added. For titration of biotinylated detection antibodies, SA-pHRP was added at the optimum concentration determined in the previous experiment. Low background was obtained with mAb A3/710 at 0.5 μg/mL and SA-pHRP at 0.5 μg/mL (Additional file 1: Figure S1).

The cutoff to determine the limits of detection (LOD) and to discern positive from negative samples was calculated as the mean + 3 × standard deviation of negative control columns (buffer only, *n* = 16). The assay’s sensitivity was determined by measuring the LOD for recombinant A27. The assay’s specificity was determined for various OPV strains by using purified viral particles and by testing clinical crust material from CPXV-infected patients. To determine the test’s specificity, additional viruses causing similar clinical pictures were used, including HSV-1 and PPV. The LOD was determined by measuring serial dilutions of semi-purified virus particles and virus from clarified cell culture supernatant. Finally, the OPV ABICAP assay was validated with a blinded panel of PCR-quantified highly pathogenic viruses, including *Yellow Fever virus*, *Ebola virus*, *Marburg virus*, VACV, and MPXV.

## Results

To establish the ABICAP assay, two high-affinity and epitope-matched monoclonal antibodies targeting the orthopoxviral attachment protein A27 were tested: mAb A1/40 and mAb A3/710. Based on previous results from sandwich-ELISAs, mAb A1/40 was immobilized as a capture ELISA while biotinylated mAb A3/710 was used as detection antibody. This combination proved superior to all other combinations tested with 8 monoclonal antibodies targeting A27 [11].

### Detection limits

**Fig. 2:**
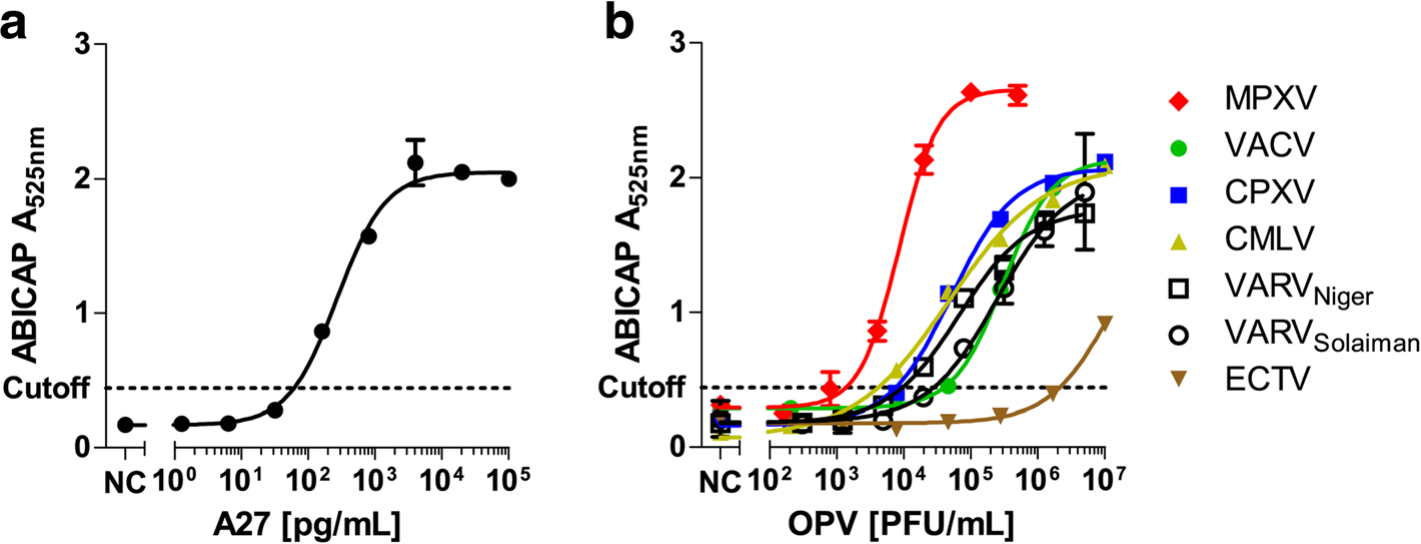
Titration of recombinant A27 proteins and different OPV strains to determine detection limit for the ABICAP assay. **a** Results of titration for recombinant A27 (BEI Resources). **b** Results of titration of *Vaccinia virus* (VACV), *Cowpox virus* (CPXV), *Camelpox virus* (CMLV), *Monkeypox virus* (MPXV), *Ectromelia virus* (ECTV), and *Variola virus* (VARV) on ABICAP columns

To determine the overall sensitivity of the ABICAP as compared to the previously established sandwich-ELISA based on the same two antibodies implemented in the ABICAP assay, we determined the LOD for recombinant A27 (Fig. 2a). Here, 56 pg/mL of recombinant A27 could be detected which is approximately ten times higher as compared to the LOD by ELISA of 5 pg/mL [11]. The higher LOD was mainly caused by the highly stringent cutoff of 0.417, which was calculated based on testing negative controls of several batches of ABICAP columns in independent experiments. If only the negative controls of the titration experiment were used to calculate the cutoff (0.234), a comparable sensitivity of 17 pg/mL was achieved.

Using the presented protocol, all OPV strains tested were detected with sensitivities of 3.4 × 10^4^ PFU/mL for VACV, 2.4 × 10^4^ and 8.3 × 10^3^ PFU/mL for VARV (Solaiman and Niger strain, respectively), 6.8 × 10^3^ PFU/mL for CPXV, 1.3 × 10^3^ PFU/mL for MPXV, and 6.8 × 10^3^ PFU/mL for CMLV (Fig. 2b). ECTV showed a significantly increased LOD (1.9 × 10^6^ PFU/mL), which was expected since a previous study showed impaired binding of capture antibody A1/40 to ECTV [11]. As ECTV does not cause zoonotic disease in humans and usually only infects laboratory mice, the lacking sensitivity is irrelevant for clinical application of the OPV ABICAP assay.

### Analysis of clinical samples

Our main goal was to establish a rapid POC diagnostics system for OPV detection. We therefore tested whether the established OPV ABICAP assay was able to detect virus particles from clinical specimens. To this aim, we used biopsy material (crust, skin biopsy, and swab of crust) from two CPXV infections which had been diagnosed as CPXV positive by qPCR. Homogenized crusts of either unknown etiology (OPV negative by PCR) or those positive for *Myxoma virus* as well as a surface swab sample positive for *Varizella zoster virus* were included to test the specificity of the ABICAP assay. Cell culture supernatants from PPV and HSV-1 infections were used as negative control. The clinical material that was tested positive for OPV by PCR could be diagnosed as positive by the OPV ABICAP assay (Table 1). Even below the stringently chosen cutoff of 0.417, the cat skin biopsy with a corresponding qPCR C_T_ value of 27.5 showed significantly higher signals than PPXV or HSV-1 used as negative control. No false-positive results were obtained for homogenized crusts of either unknown etiology or caused by *Myxoma virus*. The assay also showed no cross-reactivity against a sample positive for *Varizella zoster virus*. Most importantly, the swabbed crust sample was unambiguously positive, indicating that non-invasive sampling of crusts and rapid testing by the OPV ABICAP assay is possible, which turns the assay into a promising tool in field diagnostics.

**Table 1:** Validation of the OPV ABICAP assay with clinical samples

Sample Source	Diagnosed as	qPCR [C_T_]^a^	Tested Dilution	ABICAP^b^ [A_525nm_]	Result^c^
Cat, homogenized crust	Cowpox^d^	17.4 (1.6 × 10^9^ GE/mL)	1:50/1:250	2.398/1.033	++/++
Cat, homogenized skin biopsy	Cowpox^d^	27.5 (1.4 × 10^6^ GE/mL)	1:50/1:250	0.390/0.249	±/-
Human, homogenized crust	Cowpox^d^	11.7 (8.2 × 10^10^ GE/mL)	1:50/1:250	1.428/0.446	++/+
Human, swab of crust	Cowpox^d^	19.6 (3.4 × 10^8^ GE/mL)	1:10/1:50	1.912/0.987	++/+
Human, homogenized crust	Unknown (OPV + PPV PCR negative)	n.a.	1:50	0.334 ± 0.049	-
Rat, homogenized crust	Unknown (OPV PCR negative)	n.a.	1:50	0.245 ± 0.007	-
Rabbit, homogenized crust	Myxomavirus	n.a.	1:50	0.305 ± 0.092	-
Human, swab of rash	Varizella zoster	29.5	1:50	0.285 ± 0.007	-
Cell culture	Parapox virus	-	1:10 (2.5 × 10^5^TCID_50_/ ml)	0.275 ± 0.022	-
Cell culture	Herpes simplex virus	-	1:10 (6.9 × 10^7^ PFU/ ml)	0.235 ± 0.065	-

^a^In parentheses: estimation of genome equivalents in undiluted samples. For negative controls (Parapox virus and herpes simplex virus cell culture supernatant): titer of tested 1:10 dilutions

^b,d^For scarce sample material, tested in two dilutions (1:10 and 1:50). Anywhere else, duplicate measurements (mean ± standard deviation of *n* = 2)

^c^A_525nm_ < 0.417 (cutoff): - (negative), 0.417 < A_525nm_ < 1.0: + (positive), 1.0 < A_525nm_ :++ (highly positive). ±: borderline positive, see text

### Application of the ABICAP OPV assay in an external quality assessment for diagnostics of BT samples

To evaluate further the ability of the OPV ABICAP assay to detect OPV and differentiate them from other potential bioterror agents, a panel of PCR-quantified gamma irradiation-inactivated viruses was tested (Table 2). The panel was analyzed in a blinded fashion and was unblinded after reporting of the results. Here, VACV could be detected down to a C_T_ value of 30.7 corresponding to 6.4 × 10^4^ genome equivalents (GE)/mL. The highest dilution at a C_T_ value of 37.98 (2.3 × 10^3^ GE/mL) could not be detected, which is in agreement with the titration experiments. In addition, to our knowledge OPV preparations contain at least 10-fold higher viral genome loads than infectious particles, meaning that the particle concentration in this sample was significantly lower. MPXV was also reliably detected, while no cross-reactivity could be seen against *Ebola virus*, *Marburg virus*, and *Yellow fever virus*.

**Table 2:** Validation of the OPV ABICAP assay in an external quality assessment for diagnostics of BT samples

Virus	qPCR [C_T_]	Genome equivalents tested [GE/mL]	ABICAP^a^ [A_525nm_]	Result
Monkeypox	27.0	9.2 × 10^5^	1.570 ± 0.001	++
Vaccinia	22.5	2.4 × 10^7^	2.032 ± 0.185	++
Vaccinia	30.7	6.2 × 10^4^	0.437 ± 0.023	+
Vaccinia	38.0	2.3 × 10^3^	0.119 ± 0.016	-
Ebola	23.5	2.7 × 10^6^	0.169 ± 0.009	-
Marburg	23.3	8.2 × 10^6^	0.152 ± 0.050	-
Yellow fever	n.d.^b^	-	0.194 ± 0.004	-
Cell culture medium (DMEM)	-	-	0.181 ± 0.005	-

^a^Mean ± standard deviation of *n* = 2

^b^Not determined

In conclusion, the OPV ABICAP assay was able to detect OPV rapidly from clinical specimens as well as from blinded samples included in a bioterror panel. The assay was sufficiently sensitive to confirm the virus presence in all clinical samples. The observed LOD for real-world samples measured with the OPV ABICAP assay corresponded to approximately 5 × 10^4^ GE/mL.

## Discussion and conclusions

Molecular diagnostics of infectious diseases has undergone a rapid development over the past years. While there are numerous commercially available tests for clinically relevant infectious diseases, there is an obvious gap regarding tests for rare or emerging diseases, particularly with respect to on-site diagnostic tests. Although unrivalled in sensitivity and specificity, PCR has its drawbacks in field diagnostics, requiring a certain amount of technical equipment, practical skills, and diagnostic knowledge. In contrast, so-called rapid tests like lateral flow assays (LFA) usually circumvent these demands and are easy to perform, but lack sensitivity due to the absence of a signal amplification step. However, they present a promising approach for POC diagnostics.

The established OPV ABICAP immunofiltration assay is the first POC detection assay covering all zoonotic OPV as well as VARV with a satisfactory sensitivity. One previously developed handheld rapid test for poxviruses, the Tetracore Orthopox Biothreat detection assay, displays an LOD of 10^6^ to 10^7^ PFU/mL which is usually sufficient for the diagnosis of skin lesions containing extremely high viral loads [20]. The new OPV ABICAP assay offers an LOD as low as 10^4^ PFU/mL which allows reliable diagnosis even when viral loads in the sample are low because of inappropriate sampling or non-optimal time points in the course of infection. Similarly, an ABICAP assay for the detection of *Ebola virus* showed an LOD of approximately 1.2 × 10^4^ PFU/mL, indicating that ABICAP systems generally represent sensitive platforms for particle detection [10]. Additionally, the described LODs can be reached within 45 min, which is comparable to the Tetracore assay time.

The higher sensitivity of the ABICAP assay compared to lateral flow assays is also in agreement with previous reports with an ABICAP assay targeting botulinum neurotoxin. Furthermore, in an independent evaluation the ABICAP showed sensitivity comparable to that of an in-house ELISA whereas commercial LFAs either failed completely or lacked sensitivity, even compared to the relatively high LODs according to the manufacturers [21]. Our study also shows that well-characterized antibodies can easily be transferred from ELISA to ABICAP assays. Here, the specific characteristics of the immunofiltration approach offer the clear advantage of relative simplicity and rapid assay times at comparable sensitivity.

Particularly when performing field diagnostics, adequate sampling and sample processing can be impaired dramatically by environmental conditions. Although the ABICAP assay was highly specific for all samples tested in this work, false-positive results remain a risk as in any diagnostics, especially when testing clinical sample material. Independent of the high specificity of the implemented antibodies, matrix effects might cause higher background signals. For an exhaustive validation, more samples, ideally in the field, will be required to be analyzed using this assay.

Finally, as all reagents for an OPV ABICAP test can be stored stably at 4 °C or even ambient temperature, there is no need to freeze reagents to -20 °C, which is usually a limiting factor in the regions in which the diagnosed diseases are endemic. The OPV ABICAP assay is therefore a low-tech alternative, particularly when compared to PCR, and represents an optimal compromise between ease of handling and sensitivity. Most importantly, not only was the ABICAP system able to detect OPV from clinical samples, but also to differentiate OPV from other viruses that can (like OPV) cause skin lesions or potentially be used as bioterror agents.

## Additional file

Additional file 1: Figure S1. Titration of SA-pHRP and biotinylated detection antibody A3/710. Working concentrations for both SA-pHRP (A) and A3/710 (B) were determined by testing four concentrations (0.1, 0.5, 1, 5 μg/mL), using the ABICAP protocol to minimize background binding caused by too high antibody or enzyme concentrations (TIF 164 kb)
