# Supplementary figures and images for: Rapid and sensitive point-of-care detection of Orthopoxviruses by ABICAP immunofiltration
Source: Virol J. 2016 Dec 9;13:207. doi: 10.1186/s12985-016-0665-5 (PMC5148848; doi:10.1186/s12985-016-0665-5)

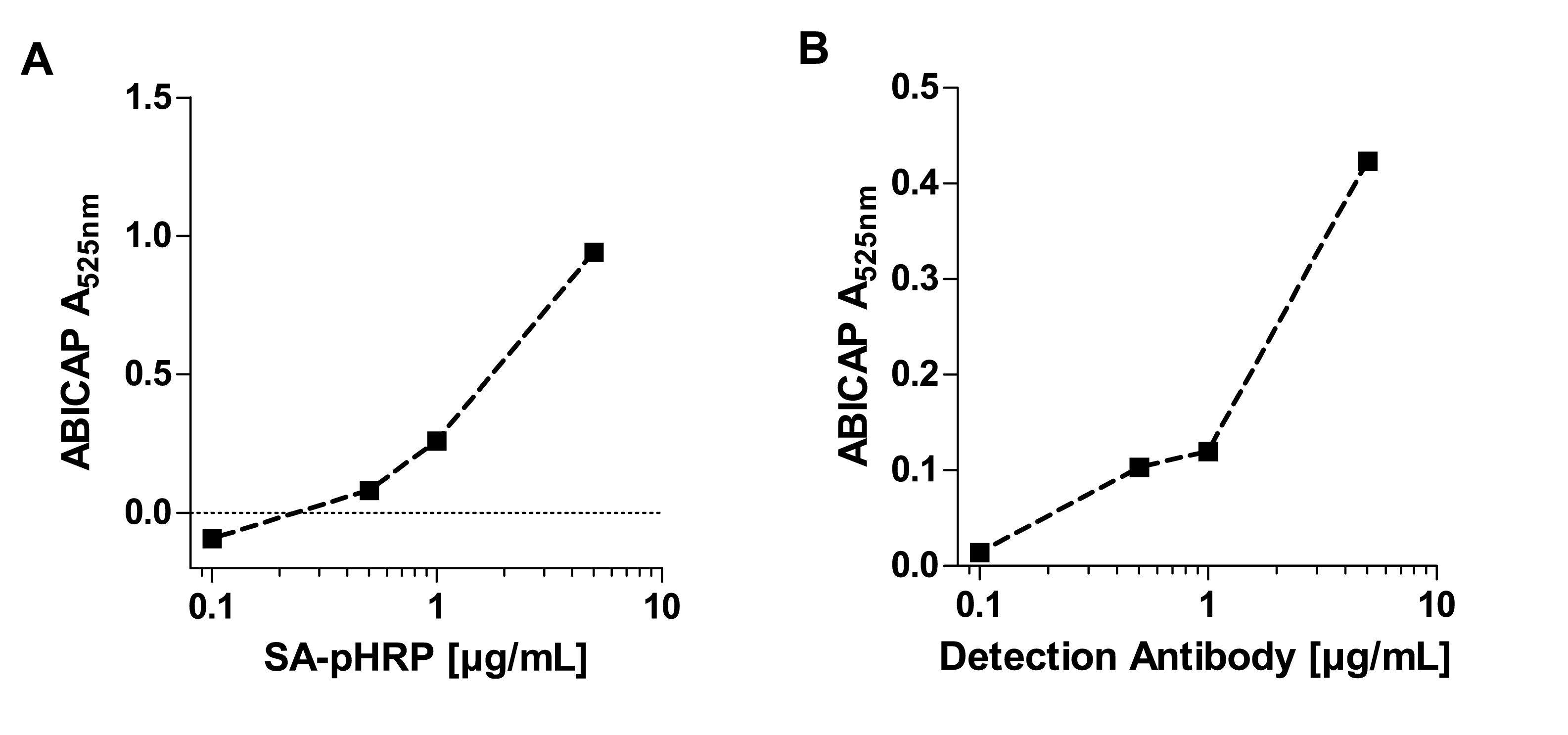

Supplement: Additional file 1: Figure S1. — Titration of SA-pHRP and biotinylated detection antibody A3/710. Working concentrations for both SA-pHRP (A) and A3/710 (B) were determined by testing four concentrations (0.1, 0.5, 1, 5 μg/mL), using the ABICAP protocol to minimize background binding caused by too high antibody or enzyme concentrations (TIF 164 kb) [file 12985_2016_665_MOESM1_ESM.tif]
